# Supplementary material for: Ultraviolet-C Irradiation, Heat, and Storage as Potential Methods of Inactivating SARS-CoV-2 and Bacterial Pathogens on Filtering Facepiece Respirators
Source: Pathogens. 2022 Jan 10;11(1):83. doi: 10.3390/pathogens11010083 (PMC8780977; doi:10.3390/pathogens11010083)
Supplement: Supplementary file 1 [file pathogens-11-00083-s001.zip › pathogens-1538918-supplementary.pdf]

Equipment

- Sewing machines
  - Elna 3003 (Geneva, Switzerland)
  - Singer Heavy Duty 4432 (La Vergne, Tennessee, USA)

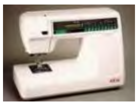 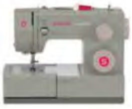

- Threads
  - Black and beige Synthetic (100% polyester) threads (Gütermann, Gutach-Breisgau, Germany)

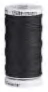 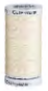

- Trimming scissors
- 8-mm drive pin punch (hole punch) and hammer

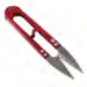 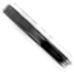 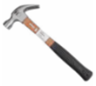

Cut, make and trim

All surface areas and equipment were cleaned and disinfected with isopropanol prior to production of coupons.

As FFRs are multi-layered, individual coupons had to be machine stitched to secure layers in place during experiments. A sewing machine uses two spools of thread to form a stich, one on the top (which threads through the needle) and one from the bottom (known as bobbin spool). For the top spool, black thread was used to indicate the outer-facing (external) surface of the mask. The bottom spool (bobbin) was wound with beige thread to indicate the inside (wearer facing side) of the mask. Synthetic threads were used (Gütermann, Gutach-Breisgau, Germany), as cotton threads are more hydrophilic and could potentially absorb liquid from the inoculum.

Note that both surfaces of the coupon were stitched at the same time with a single stitch with a reversed stitch to hold (anchor) the stitch.

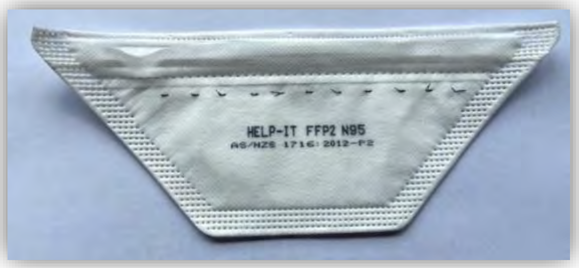

Coupons were only created using the hole puncher where the stitches cleanly 'fit' the 8-mm drive pin punch circumference.

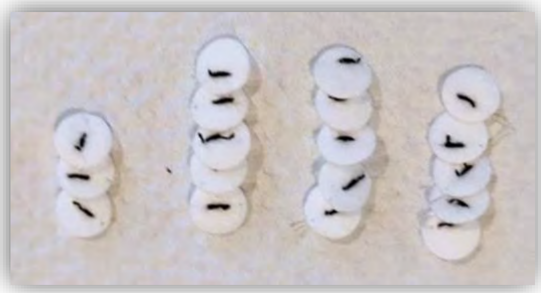

Once round coupons were punched, the excess thread was trimmed off, and the coupons were packaged.

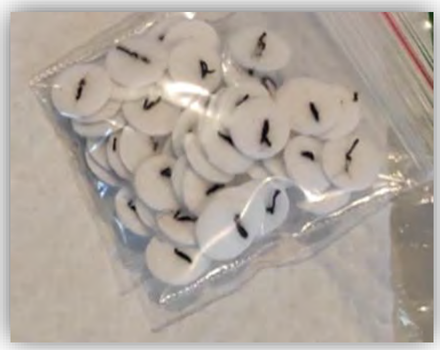

Figure S1. Preparation of Coupons from Filtering Facepiece Respirators (FFRs)

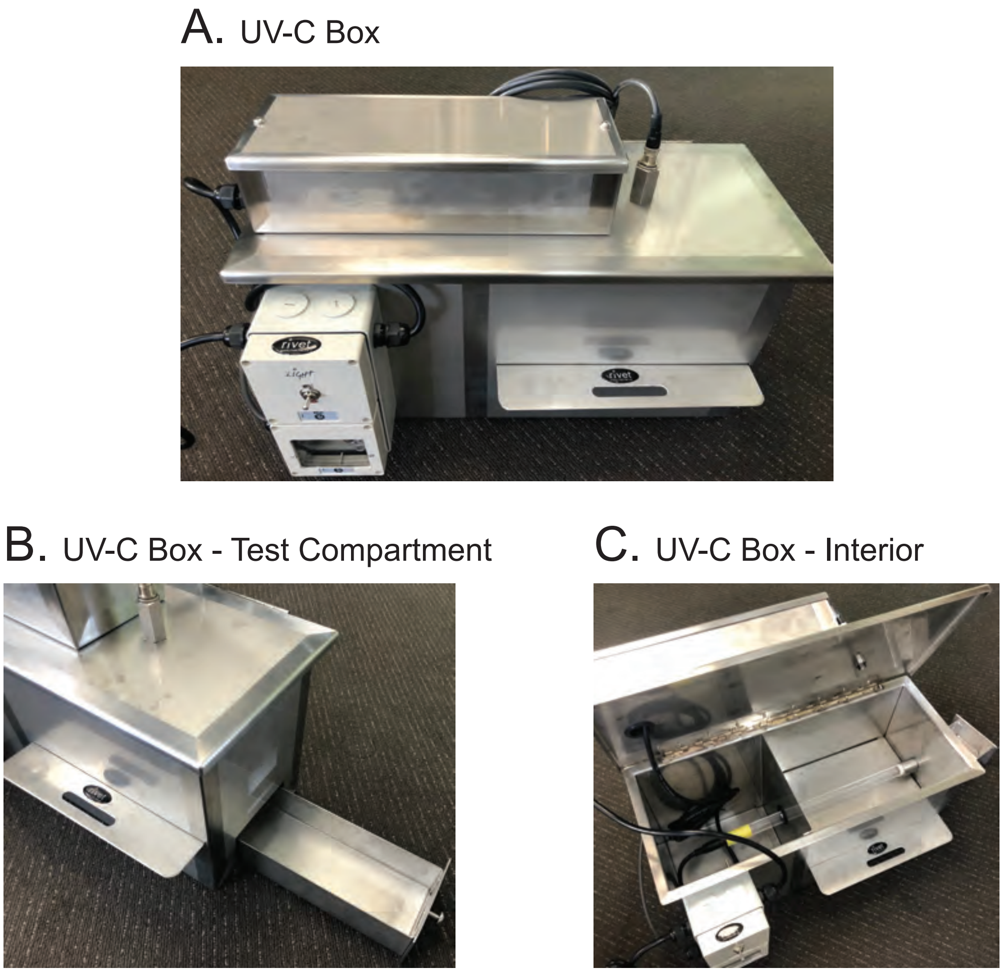

Figure S2. (**A**) Ultraviolet-C (UV-C) device specifically designed for this study by UV Solutionz (Kekrikeri, New Zealand) measuring 50 cm long x 23.5 cm wide x 26 cm high, which was designed to fit inside a Class II Biosafety Cabinet in the PC3 laboratory at the University of Otago. (**B**) Single, 27 cm long x 10 cm wide x 5 cm deep, drawer designed to accommodate a cell-culture plate lid (e.g., 24-well plate) with inoculated coupons. Once the drawer is shut, exposure to UV-C light was controlled using a metal shutter located above the drawer and below the light source. (**C**) Interior of the UV-C box showing the single UV lamp model EE4066LP with an amalgam filament, 1.8 Amp, 40V, and 72W, averaging an UV-C irradiance of 5.56 mW/cm^2 at 254 nm, within a narrow range of 0.10 mW/cm^2. The lamp is located 100 mm from the base of the drawer, where the sensor eye is placed.

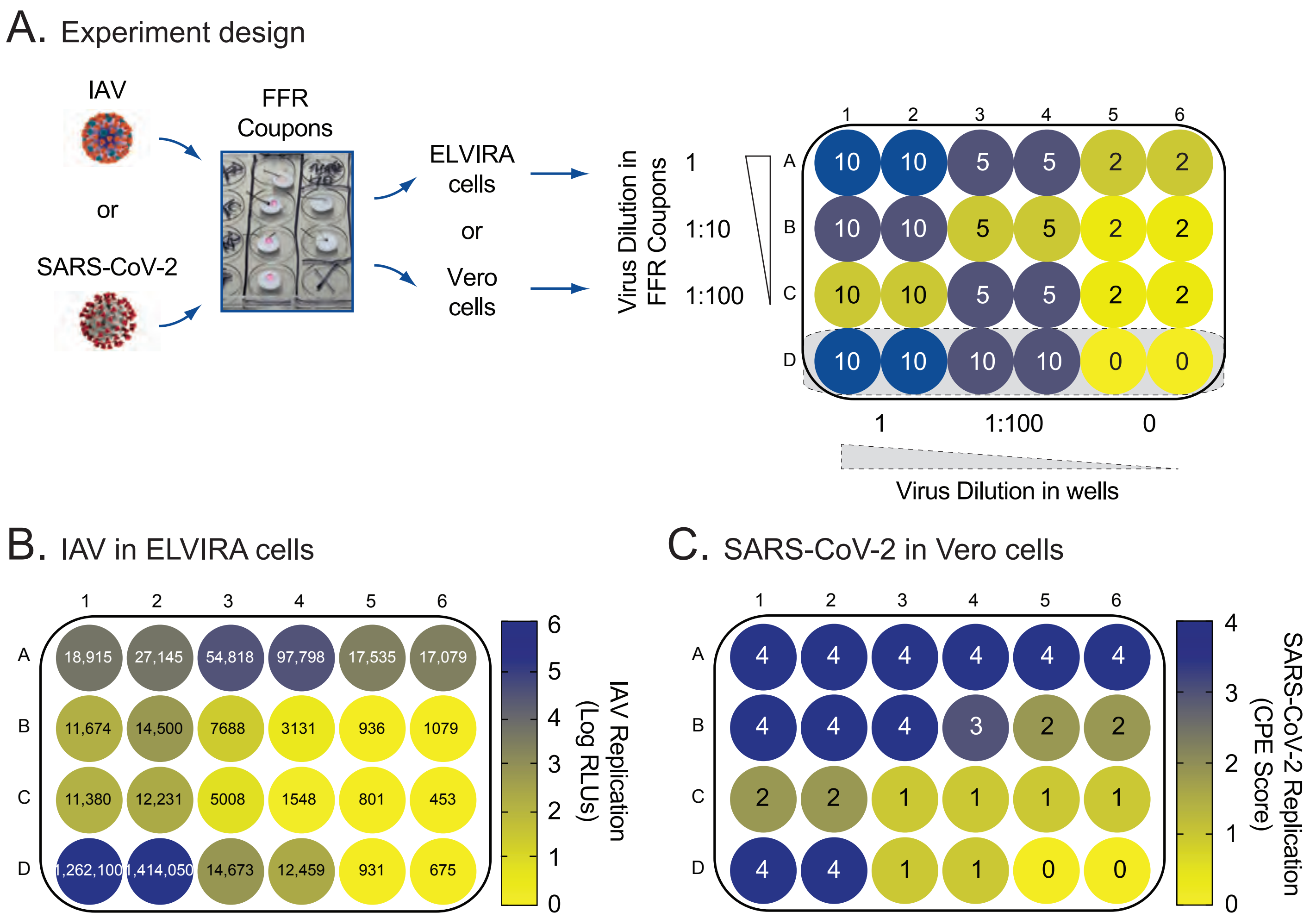

Figure S3. Pilot study to test the filtering facepiece respirator (FFR) coupon system. (**A**) FFR coupons were inoculated with 10 ul of serial dilutions (1, 1:10, or 1:100) of Influenza A virus (IAV) A/Mallard/Alberta/287/2012 (H1N1) strain or SARS-CoV-2 NZ/Queenstown/01 strain, incubated for one hour and then placed inverted into wells containing ELVIRA Flu A cells or Vero cells to quantify IAV or SARS-CoV-2 replication after one or three days, respectively. The last row of the plates corresponded to positive (columns 1 to 4, virus dilutions) and negative (columns 5 and 6, cells only) controls. (**B**) IAV replication quantified by measuring the expression of firefly luciferase in ELVIRA cells in response to IAV infection. Values correspond to relative light units (RLUs). (**C**) SARS-CoV-2 replication quantified by determining cytophatic effect (CPE) in Vero cells. CPE score: 0, no CPE; 1, <25% CPE; 2, 25 – 49% CPE; 3, 50 – 74% CPE; and 4, 75 – 100% CPE.
